# An annotated checklist of freshwater Copepoda (Crustacea, Hexanauplia) from continental Ecuador and the Galapagos Archipelago

**DOI:** 10.3897/zookeys.871.36880

**Published:** 2019-08-12

**Authors:** Paulo Henrique Costa Corgosinho, Maria Hołyńska, Federico Marrone, Luís José de Oliveira Geraldes-Primeiro, Edinaldo Nelson dos Santos-Silva, Gilmar Perbiche-Neves, Carlos López

**Affiliations:** 1 Dep. Biologia Geral. Universidade Estadual de Montes Claros (UNIMONTES). Av. Prof. Rui Braga, S/N - Vila Mauricéia, 39401-089, Montes Claros, MG, Brazil Universidade Estadual de Montes Claros Montes Claros Brazil; 2 Museum and Institute of Zoology Polish Academy of Sciences, Wilcza 64, 00-960 Warszawa, Poland Museum and Institute of Zoology Polish Academy of Sciences Warsaw Poland; 3 Dip.to STEBICEF. Università di Palermo. Via Archirafi 18. 90123, Palermo, Italy Università di Palermo Palermo Italy; 4 Laboratório de Plâncton, Coordenação de Biodiversidade, Instituto Nacional de Pesquisas da Amazônia (INPA), Av. André Araújo, 2936, 69060-000, Manaus, AM, Brazil Instituto Nacional de Pesquisas da Amazônia Manaus Brazil; 5 Laboratório de Plâncton, Departamento de Hidrobiologia – CCBS, Universidade Federal de São Carlos (UFSCar). Rodovia Washington Luís, Km 235 – CP 676, 13565-905, São Carlos, São Paulo, Brazil Universidade Federal de São Carlos São Carlos Brazil; 6 Escuela de Acuicultura y Pesquería. Facultad de Ciencias Veterinarias. Universidad Técnica de Manabí. Ciudadela Universitaria. Bahía de Caráquez. Manabí. Ecuador Universidad Técnica de Manabí Manabí Ecuador; 7 Escuela Superior Politécnica del Litoral (ESPOL), Centro del Agua y Desarrollo Sustentable, Campus Gustavo Galindo, Km 30.5 Vía Perimetral, Guayaquil, Ecuador Escuela Superior Politécnica del Litoral Guayaquil Ecuador

**Keywords:** Biodiversity, freshwater Copepoda, geographical distribution, Neotropics, species richness

## Abstract

An annotated checklist of the free-living freshwater Copepoda recorded in different regions in Ecuador (including the Amazon, the Andes, the coastal region, and the Galapagos Islands) is here provided. We revised all published records, critically evaluated the validity of each taxon and provided short taxonomic and biogeographical remarks for each one. A total of 27 taxa have been reported, including species and records at the generic level only. The species and taxa identified only up to the generic level belong to five families and 14 genera. The Cyclopoida is the most diverse group with 16 records belonging to species (or identified to the generic level only) and eight genera, followed by the Harpacticoida with six species, one identification to the generic level only, and four genera, and Calanoida with four species belonging to two genera. A total of 18 taxa are recorded for the Andes. Six have been recorded in the Amazon, two are recorded for the coastal region, and six for the Galapagos. One species is shared between the Amazon and the Andes. One species is shared between the coastal region and the Amazon. Seventeen are only reported from the Andes and four are only reported from the Amazon. At the current status of the knowledge, any attempt to analyze and generalize distributional patterns of copepods in Ecuador is premature due to the scarcity of available information, and evidently there is an urgent need for more extensive field collections. A few working hypothesis for future studies are identified.

## Introduction

Probably the first published studies on the Copepoda from the Neotropical region are those by Richard (1895, Haiti; 1897, South America), Sars (1901, South America), and Stingelin (1904a, 1904b). The region remained for a long time understudied, with a few taxonomic works realized in the first four decades of the 20^th^ century (e.g., Wierzejski 1892; Daday 1902; Thiébaud 1914; Brehm 1924; Kiefer 1926; Pesta 1927; Wright 1927; Delachaux 1928; Lowndes et al. 1934). From there on, after a gap of almost two decades both faunistic and taxonomic studies became more common (e.g., Noodt 1965; Brandorff 1977; Paggi 1978; Löffler 1981; Collado 1983; Dussart 1984; Reid 1984 and 1985; Santos-Silva et al. 1989, Rocha and Sendacz 1996; Corgosinho and Martínez Arbizu 2005; Perbiche-Neves et al. 2014a). Nowadays, about 561 species of Copepoda are known for the Neotropical region (Boxshall and Defaye 2008). The most diverse families are Cyclopidae (174), Canthocamptidae (109), Diaptomidae (82), and Parastenocarididae (65) (approximate number of species is within parentheses). The calanoid and cyclopoid fauna is relatively well known for the Neotropical region. As for the Harpacticoida, despite recent advances in taxonomy and zoogeography of the Parastenocarididae (e.g., Corgosinho and Martínez Arbizu 2005; Corgosinho et al. 2010), there is still much to explore, especially in the families Canthocamptidae and Parastenocarididae. Moreover, our knowledge on inland water copepod diversity is also quite unevenly distributed geographically, and most data refer to Argentina, Brazil, Colombia, and Venezuela, whereas other countries are inadequately known.

Similarly to the freshwater Cladocera and Rotifera (López et al. 2018a, 2018b), our knowledge of the Copepoda of Ecuador in comparison to other countries in tropical South America is relatively recent and very limited. This is in sharp contrast to the great habitat diversity in the country, ranging from Amazon rainforest, including uphills and the lowlands, to alpine tundra paramo (more than 4000 m a.s.l.) and to the inclusion of Ecuador as a hotspot of biodiversity for plant and vertebrate species (e.g., Myers et al. 2000; Brummitt and Lughada 2003; Rieckmann et al. 2011).

As part of an ongoing project dedicated to collecting and revising the Copepoda, Cladocera and Rotifera from inland water bodies of Ecuadorian mainland and the Galapagos Islands, we assembled a list of the inland water Copepoda known to date for the country and provide a short discussion of relevant nomenclatural issues and known geographic distribution of the species. Our goal is to identify the major information gaps and pave the way for future studies on the Ecuadorian freshwater copepods, which ultimately might allow better framing of the copepod fauna of Ecuador in the Neotropical region and understanding its origin and affinities.

## Methods

The list of the copepods of continental Ecuador and Galapagos Islands compiled herein is based on literature data, including theses and taxonomic and ecological publications. The current valid species names and combinations are mostly based on Dussart and Defaye (2002, 2006) and the WoRMS database (http://www.marinespecies.org). Here we adopt the classifications of Boxshall and Halsey (2004) and Khodami et al. (2017), who have included the Poecilostomatoida families within Cyclopoida.

**Figure 1.**
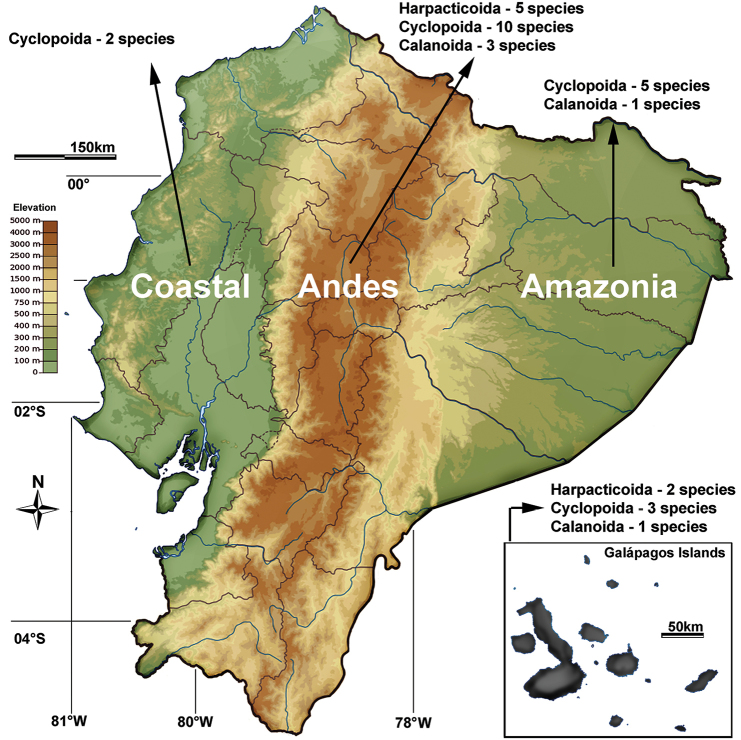
Map of Ecuador showing main geographical regions and number of recorded species for freshwater Cyclopoida, Harpacticoida, and Calanoida.

The geographic distribution of the freshwater taxa within the country is described by dividing continental Ecuador into three subregions (Andean, coastal, and the Amazonian subregions; see Steere 1950) to which the Galapagos Islands are to be added (Fig. 1). References to other regions within South America follow the biogeographical classification proposed by Dussart (1984).

Abbreviations used in the text: **enp1–3** first to third endopodal segment; **exp1–3** first to third exopodal segment; **P1–P5** first to fifth legs.

## Results

Twenty-seven records have been reported in literature from the inland water bodies of Ecuador, including the Galapagos Islands. The species and taxa identified only up to the generic level belong to five families and 14 genera. The Cyclopoida is the most diverse group with 16 records belonging to species (or identified to the generic level only) and eight genera, followed by the Harpacticoida with six species, one identification to the generic level only, and four genera, and Calanoida with four species belonging to two genera (Table 1). Eighteen taxa are recorded for the Andes, and six for the Amazon. One cyclopoid species is shared by the Amazon and the Andes. One cyclopoid species is shared between the coastal region and the Amazon. Seventeen are restricted to Andes and 4 confined to Amazon. Two species are recorded for the coastal region, and six to the Galapagos Islands.

**Table 1.** Distribution of the taxa in the four geographical regions of Ecuador. “×” indicates the occurrence of a calanoid “resembling *Notodiaptomus amazonicus*” from Lake El Junco in San Cristobal island.

Taxon	Amazon	Andes	Coastal	Galapagos
**Harpacticoida, Canthocamptidae**				
Attheyella (Chappuisiella) pichilafquensis		•		
Attheyella (Delachauxiella) freyi		•		
*Bryocamptus* sp.		•		
*Cletocamptus axi*				•
*Cletocamptus ecuadorianus*		•		
*Cletocamptus schmidti*				•
*Elaphoidella humboldti*		•		
**Cyclopoida, Cyclopidae, Cyclopinae**				
*Acanthocyclops robustus*		•		
*Acanthocyclops vernalis*			•	
*Mesocyclops meridianus*	•		•	
*Metacyclops* sp.		•		
*Metacyclops leptopus leptopus*		•		
*Metacyclops mendocinus*		•		•
*Microcyclops* sp.				•
*Microcyclops alius*	•	•		
*Microcyclops anceps*	•			
**Cyclopoida, Cyclopidae, Eucyclopinae**				
*Eucyclops agilis*		•		•
*Eucyclops breviramatus*		•		
*Eucyclops serrulatus*		•		
*Macrocyclops albidus*	•			
*Paracyclops chiltoni*		•		
*Paracyclops hardingi*		•		
**Cyclopoida, Ergasilidae**				
*Ergasilus* sp.	•			
**Calanoida, Centropagidae**				
*Boeckella gracilis*		•		
*Boeckella occidentalis*		•		
**Calanoida, Diaptomidae**				
*Notodiaptomus amazonicus occidentalis*	•			×
*Notodiaptomus cannarensis*		•		

### Harpacticoida M. Sars, 1903

#### Canthocamptidae Brady, 1880

##### Attheyella (Chappuisiella) pichilafquensis Löffler, 1961

**Distribution.** Andes (Löffler 1963).

**Remarks.** According to Löffler (1963) the type locality lies somewhere between the towns of Villarrica and Llanquihue (straight-line distance between the towns, 227 km), in the southern Chile. The color is distinctly violet and the length of the specimens from the type locality varies between 370–560 μm for males and 400–700 μm for females. In Ecuador, the specimens were larger, the females reaching a length of 900 μm and the males 700 μm. The Ecuadorian males are variable in the armature of the endopodite in P2 and P4.

##### Attheyella (Delachauxiella) freyi Löffler, 1963

**Distribution.** Andes (Löffler 1963).

**Remarks.** Originally described from Ecuador. Of the studied males and females, Löffler (1963) found that the P2enp and the P4enp can be asymmetric in armature. The dorsal ornamentation of the urosome is also variable in the male, and it can be either dorsally absent or present on the 2^nd^ to 4^th^ urosomites. Males measure are 540–700 μm long, and females are 800–980 μm long. This species was found in a high mountain pond in the southern Colombian Andes (Gaviria and Defaye 2012).

##### *Bryocamptus* Chappuis, 1929

**Distribution.**Torres and Rylander (2006) mentioned *Bryocamptus* from Ecuadorian highland lakes. However, this genus is basically boreal, with a few representatives known from New Zealand (Reid 1993), and a single species, Bryocamptus (Bryocamptus) campaneri (Reid 1993), from Central Brazil. Records of *Bryocamptus* from a lake in the state of Rio de Janeiro (Reid and Esteves 1984) are a misidentification of Attheyella (Chapuisiella) fuhrmanni (Thiébaud, 1914) (Reid 1993). *Bryocamptus broiensis* Rocha and Matsumura-Tundisi, 1976 described for the state of São Paulo is recognized by Reid (1993) as Attheyella (Delachauxiella) broiensis Reid, 1994. According to Löffler (1972), the North American species of *Bryocamptus* do not occur south of the northern limit of the Eocene-Miocene submergence of Central America.

##### *Cletocamptus axi* Mielke, 2000

**Distribution.** Collected from lagoons of the islands of Santa Cruz and Floreana, Galapagos Archipelago. Mielke (2000) referred to the type locality as “Floreana: lagoon behind the beach”.

##### *Cletocamptus ecuadorianus* Löffler, 1963

**Distribution.** Andes (Löffler 1963, as *C. deitersi ecuadorianus*).

**Remarks.** Originally described from Ecuador. Length of males reaching 620 μm long, females 750 μm long. Asymmetry is observed in the armature of the female P5 basoendopod. Both males and females show variability in the armature of the antennal exopodite. In males, P3exp-3 may be variable in armature. *C. deitersi* (Richard, 1897) has been recorded from Ecuador (Löffler 1963 as *C. deitersi ecuadorianus*), Venezuela (Escaravange and Castel 1989), Peru and Bolivia (Harding 1955), Haiti (Kiefer 1936), and USA (California; Dexter 1995). However, several authors (Dexter 1995; Suárez-Morales et al. 1996; Gee 1999; Mielke 2000, 2001) have suggested that *C. deitersi* consists of a number of morphologically indistinguishable sibling species (Gómez 2005). According to Gómez et al. (2004), *C. deitersi* is a species inquirenda, because Richard’s (1897) original description is based on highly conservative features that are not useful for species separation. Future study of specimens from all of these localities is required to show if the records refer to *C. ecuadorianus* or to *C. deitersi*. Both species are in need of redescription, and *C. ecuadorianus* is considered to be a species inquirenda.

##### *Cletocamptus schmidti* Mielke, 2000

**Distribution.** Collected from lagoons of the islands of Santa Cruz, Galapagos. Type locality, Laguna de Puerto Núñez.

**Remarks.** According to Mielke (2000), *C. axi* and *C. schmidti* slightly differ from each other in their body ornamentation and in the chaetotaxy of the exopodites of P3 and P4. Although Mielke (2000) have provided a very detailed illustration of both *C. axi* and *C. schmidti* and a complete description of their anatomical details, Wells (2007) considered *C. axi* a species inquirenda but without giving an explanation. Both species fit well the range of variability of *C. deitersi*, yet co-occurrence of the two morphotypes and the lack of intermediate forms support that *C. axi* and *C. schmidti* are separate species rather than morphological variants of a single species (Mielke 2000).

##### *Elaphoidella humboldti* Löffler, 1963

**Distribution.** Andes (Löffler 1963).

**Remarks.** Originally described from Ecuador. The male reaches 620 μm and the female is unknown.

According to Gaviria and Defaye (2015: 1026) the “Diversity of *Elaphoidella* Chappuis, 1929 in Colombia (5 species) is lower than in Cuba (10) and Brazil (9), but higher than in Suriname (2) and Argentina (2). However, these data are not the result of extensive research and sampling of all biomes and environments. Thus, we cannot draw any biogeographical pattern from this study. Only one species is known from each of the following Neotropical countries: Mexico, Costa Rica, Venezuela, French Guiana, Ecuador, Peru and Paraguay. The French islands Bonaire and Martinique are also inhabited by one species each.” Groundwater, benthic habitats of high Andean lakes, and aquatic habitats within rainforests are potential habitats for harpacticoid copepods and particularly for *Elaphoidella* Chappuis, 1929. Other still poorly investigated biotopes are phytotelmata and semiterrestrial habitats, which would no doubt yield new species of copepods (Gaviria and Defaye 2015).

### Cyclopoida Burmeister, 1834

#### Cyclopidae Rafinesque, 1815

##### Cyclopinae Rafinesque, 1815

###### *Acanthocyclops robustus* (G.O. Sars, 1863)

**Distribution.** Andes (surroundings of Antisana volcano >3000 m a.s.l.) (Löffler 1963).

**Remarks.**Löffler (1963) noted that all *Acanthocyclops* Kiefer, 1927 specimens from Ecuador possessed a spine formula of the “*vernalis* type” (2.3.3.3). However, two, three, three, and three spines on the terminal exopodal segments on P1 to P4, respectively, may occur in both *A. robustus* and *A. vernalis* Fischer, 1853, which are currently considered distinct from one another.

*Acanthocyclops robustus* is supposedly restricted to the northern Holarctic region (Mirabdullayev and Defaye 2002). All records of *A. robustus* from the southern hemisphere need verification, although introduction outside the native range by human activities cannot be excluded. The morphology and taxonomic relationships of *A. robustus* have been revised by Mirabdullayev and Defaye (2002, 2004), but see Miracle et al. (2013) for an alternative opinion on the taxonomy of the *A. robustus* group.

The genus is most diversified in the northern temperate region. So far reported from South America, there are only two species, here not including the southern South American *A. michaelseni* (Mrázek, 1901) and *A. skottsbergi* Lindberg, 1949 for which the generic affinities of which are still under debate. However, a few species ,which are apparently closely related to the *A. vernalis-robustus* group, have been described from Mexico (*A. rebecae* Fiers & Ghenne, 2000, *A. caesariatus* Mercado-Salas & Suárez-Morales, 2009, *A. marceloi* Mercado-Salas & Suárez-Morales, 2009) and Honduras (*A. smithae* Reid & Suárez-Morales, 1998); the geographic distribution is still poorly understood of these species . The occurrence of *Acanthocyclops* in South America may raise intriguing questions of the taxonomic identity and evolutionary origin of these taxa.

###### *Acanthocyclops vernalis* (Fischer, 1853)

**Distribution.** Coastal (Quimi 2014).

**Remarks.** This species, which was originally described from the neighborhood of St Petersburg, Russia (Fischer 1853), needs redescription. The actual distributional area is likely confined to the Palearctic region (Einsle 1996), and all South American records need verification (see also *A. robustus*).

###### *Mesocyclops meridianus* (Kiefer, 1926)

**Distribution.** Coastal and Amazon (Napo river valley) (Löffler 1963).

**Remarks.** The species range is likely restricted to South America. *Mesocyclops meridianus* (Kiefer, 1926), which was described from San Bernardino, Paraguay, is morphologically highly similar to *M. pseudomeridianus* Defaye & Dussart, 1988 (type locality: Mana, French Guiana), *M. brasilianus* Kiefer, 1933 (type locality: Manaus, Amazon), *M. varius* Dussart, 1987 (type locality: Taxisco, Guatemala], *M. venezolanus* Dussart, 1987 (type locality: Lake Valencia, Venezuela), and *M. meridionalis* Dussart & Frutos, 1985 (type locality: Corrientes, Argentina) (Hołyńska et al. 2003). Some older records of *M. meridianus*, therefore, might refer to other representatives of the *meridianus*-complex. The native range of the *meridianus*-clade (Hołyńska 2006) is confined to South- and Central America, as far as the Isthmus of Tehuantepec. Hołyńska (2006) hypothesized that *M. pseudomeridianus* and *M. brasilianus* are junior synonyms of *M. meridianus* and that *M. varius* is a junior synonym of *M. venezolanus*. She also emphasized the need to examine the topotypes of taxa (e.g., *M. brasilianus*, *M. meridianus*, and *M. varius*) with old or scarce original material to resolve possible synonymies. The *meridianus*-*brasilianus*-*pseudomeridianus* lineage (=? *M. meridianus*) and the *venezolanus-varius* lineage (=? *M. venezolanus*) differ from each other in the shape of the lateral arms of the seminal receptacle (Hołyńska et al. 2003). Gutiérrez-Aguirre and Suárez-Morales (2003) and Gutiérrez-Aguirre et al. (2006) presented another view of the taxonomic relationships of these and considered *M. meridianus* and *M. brasilianus* to be distinct species and put *M. varius* and *M. venezolanus* in synonymy with *M. brasilianus*.

The genus is distributed worldwide and is represented by 13 native species in South America, most of which (10 of 13) are endemic to the continent. This number included *M. aspericornis* (Daday, 1906) but excludes *M. ogunnus* Onabamiro, 1957, which is a supposedly recently introduced species. More extensive sampling will likely reveal more species in Ecuador.

###### *Metacyclops* Kiefer, 1927

**Distribution.***Metacyclops* are the dominant cyclopoid taxa in glacial lakes in the tropical Andes (Van Colen et al. 2017).

###### *Metacyclops leptopus leptopus* (Kiefer, 1927)

**Distribution.** Glacial lakes, 3800–4000 m a.s.l. in Páramo de Guamaní, Andes (Torres and Rylander 2006).

**Remarks.***Metacyclops leptopus leptopus* was originally described from Lake Huarón and Lake Naticocha in Region Pasco in the High Andes of Peru (Kiefer 1926, 1927). Currently four subspecies are distinguished: *M. leptopus leptopus* (high-altitude lakes in Bolivia, Colombia, Ecuador, Peru, and possibly Venezuela; Reid et al. 1990; Dussart and Defaye 2006; Gaviria and Aranguren 2007); *M. leptopus mucubajiensis* Kiefer, 1956 (Laguna de Mucubaji, Venezuelan Andes, 3620–3650 m a.s.l.); *M. leptopus totaensis* Reid, Arevalo & Fukushima, 1990 (Lago de Tota, Colombian Andes, 3015 m a.s.l.); and *M. leptopus venezolanus* Kiefer, 1956 (Mariposa Reservoir, Caracas, Venezuela, ca 985 m a.s.l.). The latter subspecies was considered by Dussart (1984) and Dussart and Defaye (2006) to represent *M. mendocinus* rather than a lineage within *M. leptopus*. For more comments on the taxonomic relationships of *M. leptopus*, see *M. mendocinus*.

###### *Metacyclops mendocinus* (Wierzejski, 1892)

**Distribution.**Löffler (1963) reported this species from numerous sites in the Andes, and Steinitz Kannan (1979) found it in Lake Cuicocha, Chicapan (= San Pablo), and Yaguarcocha. Peck (1994) reported it in the in the Galapagos from temporary pools on Isla Santa Cruz.

Originally described from northern and western Argentina (Jujuy and Mendoza Provinces) (Wierzejski 1892), this species is widely distributed in both South America (Bolivia, Brazil, Chile, Colombia, Paraguay, Peru, Uruguay, and Venezuela), and Middle America (Cuba, Haiti, Nicaragua, and Puerto Rico) (Dussart and Defaye 2006).

**Remarks.** The remote mid-Atlantic islands of the Azores harbour a subspecies, *M. mendocinus insularis* Defaye & Dussart, 1991, which suggests that this species has good capacity for dispersal. *Metacyclops mendocinus*, along with *M. leptopus*, belongs to a group of species that are predominantly Neotropical in distribution. They share the 12-segmented state of the antennule and two terminal spines on the terminal endopodal segment of P4 (Herbst 1988). The relationships of *M. mendocinus* to the *M. leptopus*-complex need to be revised. Reid et al. (1990) proposed the use of the relative length of the inner terminal caudal (longest) seta as the main distinguishing character between *M. mendocinus* (seta less than twice as long as caudal ramus) and members of the *M. leptopus*-complex (seta 2.6 or more times longer than caudal ramus). Reid et al. (1990) also mentioned ecological differences between the two species: *M. mendocinus* appears to be eurytopic, while *M. leptopus* apparently inhabits relatively pristine lakes at mostly high altitudes. Accordingly, the records from Andean Ecuador might refer to *M. leptopus* rather than *M. mendocinus* (Reid et al. 1990).

###### *Microcyclops* Claus, 1893

**Distribution.**Peck (1994: 57) mentioned the occurrence of a *Microcyclops* sp. (“probably a native species”), inhabiting temporary freshwater pools in Isla Santa Cruz, tortoise reserve (120 m a.s.l) in the Galapagos Archipelago. Species of *Microcyclops* were the dominant Cyclopoida in glacial lakes in the tropical Andes (Van Colen et al. 2017).

###### *Microcyclops alius* (Kiefer, 1935)

**Distribution.** Andes (Lake San Pablo, Imbabura Province in northern Ecuador; 2700 m a.s.l.) and Amazon (Napo river valley) (Löffler 1963).

**Remarks.** This species was originally described from Santa Lucia, Southern Uruguay. Rocha (1998) supposed that *M. alius* is a junior synonym of *M. dubitabilis* (Kiefer, 1934) (type locality: Trou Caiman Lake, near Port au Prince, Haiti). In a revision of the American *Microcyclops*, Gutiérrez-Aguirre and Cervantes-Martínez (2016) confirmed the conspecificity of these taxa, and for a redescription of *M. dubitabilis* (Kiefer 1934) (= *M. alius*), see Gutiérrez-Aguirre and Cervantes-Martínez (2016). The geographic range of *M. dubitabilis* stretches from Florida Keys, USA (Reid and Hribar 2006) through Mexico, Central America, and the Caribbean islands to South America, as far as possibly the middle Paraná River, Argentina) (Dussart and Defaye 2006).

The genus, which has approximately 54 species or subspecies, is distributed worldwide, yet most diversified in the tropics, where there are 42 species. South America harbours about 12 species, and we expect more taxa occur in Ecuador.

###### *Microcyclops anceps* (Richard, 1897)

**Distribution.** Amazon (Napo river valley) (Löffler 1963).

**Remarks.** The type locality is Rio Grande do Sul, Brazil (Richard 1897). Two subspecies are distinguished. The range of the nominotypical subspecies extends from Mexico throughout Central and South America as far as Chubut Province, Argentina (Menu-Marque 2001; Dussart and Defaye 2006). *Microcyclops anceps pauxensis* Herbst, 1962 is known from its type locality at Lago Pauxís in the Brazilian Amazon. Reid (1985) synonymized the form M. anceps var. minor (Dussart 1984) from the Unaré river valley, northern Venezuela with the Amazonian *M. anceps pauxensis*. Given the current knowledge of the morphology of the American *Microcyclops*, and *M. anceps* s. s. in particular (see Gutiérrez-Aguirre and Cervantes-Martínez 2016), the taxonomic position of *M. anceps pauxensis* and the Venezuelan form need to be revised, as they may represent distinct species rather than subspecies of *M. anceps*.

##### Eucyclopinae Kiefer, 1927

###### *Eucyclops agilis* (Koch, 1838)

**Distribution.** Galapagos Islands (Isla Santa Cruz), temporary pools, 120 m a.s.l. (Peck 1994); Andes (Lake Cunro, Imbabura Province), as “a cyclopoid resembling *Eucyclops agilis*” (Steinitz Kannan 1979).

**Remarks.***Eucyclops agilis* (*Cyclops agilis* in original combination), which has as its type locality Regensburg, Germany, is a nomen dubium, and its use should be avoided (Alekseev et al. 2006). In the past, the name *E. agilis* was often applied to *E. serrulatus*-like copepods, and in the Americas some of these records might refer to *E. pectinifer* (Cragin, 1883) (Dussart and Defaye 2006). The identity of *Eucyclops* Claus, 1893 from the Galapagos and Lake Cunro in the Andes need verification.

###### *Eucyclops breviramatus* Löffler, 1963

**Distribution.** Andes (Löffler 1963).

**Remarks.** The terra typica of this species is the Lake Papallacta region in the Ecuadorian Andes (3920 m a.s.l.). The general distribution of this species is poorly understood. Records from Mexico are instead another species (Mercado-Salas et al. 2016).

###### *Eucyclops serrulatus* (Fischer, 1851)

**Distribution.**Löffler (1963) reported this species from numerous sites in the Ecuadorian Andes, and Steinitz Kannan (1979) identified a cyclopoid as probably this species from Lake Yambo, Cotopaxi Province. However, these records likely refer to other species; in fact, all records of *E. serrulatus* from the Americas need verification. Alekseev et al. (2006) revised the taxonomy of this species based on classic morphological characters and integumental pore pattern. In a geographically large-scale overview of the *E. serrulatus*-complex, Alekseev and Defaye (2011) found *E. serrulatus* s. s. to be restricted to the Palearctic region. Mercado-Salas et al. (2016), in revising the Mexican fauna, failed to find *E. serrulatus*, which provides further support that the native range of this species does not include the New World. Mercado-Salas et al. (2016) demonstrated the diagnostic value of several previously overlooked morphological structures (i.e., the surface ornamentation of P4 and antennal coxobasis) in the American *Eucyclops*.

###### *Macrocyclops albidus* (Jurine, 1820)

**Distribution.** Amazon (Napo river valley) (Löffler 1963).

**Remarks.***Macrocyclops albidus* s. s. is considered to be cosmopolitan (but see Karanovic and Krajicek 2012) and have been reported from several countries in South America, including Argentina, Chile, Colombia, Ecuador, possibly Paraguay, Uruguay, and Venezuela (Löffler 1981; Reid 1985; Rocha and Botelho 1998; Dussart and Defaye 2006; Gaviria and Aranguren 2007). The other South American subspecies, *M. albidus principalis* Herbst, 1962, differs from the nominal subspecies, among others, in the full development of the inner distal seta on the terminal endopodal segment of P4 (seta reduced to short element in *M. albidus* s. s.). *Macrocyclops albidus principalis* is endemic to the Brazilian Amazon, Venezuela, and Colombia (Herbst 1962; Dussart and Defaye 2006; Gaviria and Aranguren 2007) and might perhaps represent a distinct species rather than subspecies. Löffler (1963) reported *M. albidus* from Ecuador without reference to a subspecific name. He noted that *M. albidus* did not occur in the High Andes.

###### *Paracyclops chiltoni* (G.M. Thomson, 1883)

**Distribution.** Reported by Löffler (1963) as *Paracyclops fimbriatus chiltoni* from the Andes (surroundings of the Antisana volcano).

**Remarks.** This is one of the few truly cosmopolitan species in the Cyclopidae (Karaytug 1999). *Paracyclops chiltoni* also occurs in remote islands, such as New Zealand (terra typica), the Azores in the Atlantic, Crozet and Amsterdam islands in the southern Indian Ocean, and Tahiti and Easter Island in the Pacific (Lindberg 1958; Karaytug and Boxshall 1998b). This suggests that this species could also occur in the Galapagos Islands.

###### *Paracyclops hardingi* Karaytug & Boxshall 1998

**Distribution.**Löffler (1963) reported this species, as *Paracyclops fimbriatus andinus* Lindberg, 1957, from the Ecuadorian Andes (surroundings of the Antisana volcano).

**Remarks.** The valid name is *P. hardingi* for the *Paracyclops* originally described by Lindberg (1958) from Peru and also reported by Löffler (1963) from Ecuador. *Paracyclops fimbriatus andinus* Lindberg, 1957 is a junior homonym of *P. andinus* Kiefer, 1957, and *P. hardingi* was proposed as a replacement name (Karaytug and Boxshall 1998a). Outside of Ecuador, *P. hardingi* is known from several localities in the High Andes of Peru: Lake Conococha (Ancash) and Lake Huampucocha (Junín) (the type localities of *Paracyclops fimbriatus andinus*), as well as from various water bodies near Lake Titicaca (Karaytug and Boxshall 1998a).

#### Ergasilidae von Nordmann, 1832

##### Ergasilinae von Nordmann, 1832

###### *Ergasilus* sp.

**Distribution.** Amazon (Napo river valley) (Löffler 1963).

**Remarks.**Ergasilidae are parasitic copepods, parasitizing mainly freshwater sometimes marine coastal fish. The overwhelming majority of the South American ergasilid species (69 of 75) are known from Brazil (Marques et al. 2017).

### Calanoida G.O. Sars, 1903

#### Centropagidae Giesbrecht, 1893

##### *Boeckella gracilis* (Daday, 1902)

**Distribution.** Andes (Löffler 1963; Gaviria 1989). It also occurs in the Patagonian and Paranean zoogeographic zones (Dussart 1984; Bayly 1992; Dussart and Defaye 2002).

**Remarks.** According to Löffler (1963), in spite of the numerous collections in Peru, this species has so far been found around the Titicaca Lake in the south of the country. Conversely, it is quite common and widespread in Chile and Argentina, especially in southern areas. Gaviria (1989) found this species in the Cordillera Oriental of the Colombian high Andes. The Ecuadorian population is variable in the segmentation and setation of the female P5 endopodite, which has an asymmetrically distally fused segment in some specimens, and both endopodites have one seta less. In some cases, both P5 endopodites are 2-segmented. Additionally, the left and right endopodite of the male is also variable in length, shape, and segmentation. Such pronounced variability is worthy of further study.

##### *Boeckella occidentalis* Marsh, 1906

**Distribution.** Andes (Löffler 1963, Brehm 1924; Delachaux 1928, as *Pseudoboeckella godeti*; Dussart 1984; Bayly 1992; Gaviria 1989; Dussart and Defaye 2002; Van Colen et al. 2017).

**Remarks.** According to Löffler 1963, the Ecuadorian populations of this species, which is abundant in the Peruvian Andean regions, differ slightly from the type as described by Marsh (1906). The enp-3 of the female P5 bears six setae. In the male, the P5 is very similar to the drawings provided by Delachaux (1928), Harding (1955), and Löffler (1955). In both the Peruvian and Ecuadorian populations the distal portion of the right P5 endopodite is recurved, scythe-shaped, and with tuberculi along its outer margin (each tuberculum with a small seta). Gaviria (1989) found this species in the Cordillera Oriental of the Colombian high Andes.

Torres and Rylander (2006) and Araujo et al. (2014) mentioned the subspecies *Boeckella occidentalis intermedia*, yet neither WoRMS (2019) nor Dussart and Defaye (2002) include this taxon. This is probably not a valid name.

#### Diaptomidae Baird, 1850

##### *Notodiaptomus amazonicus occidentalis* Löffler, 1963

**Distribution.** Amazon (Löffler 1963).

**Remarks.**Löffler (1963) described the subspecies *N. amazonicus occidentalis* based on a few mature males collected in the Napo river valley of northeastern Ecuador. No mature females were available to Löffler, so that the morphology of the female is currently unknown.

According to Löffler (1963), this taxon is so closely similar to *N. amazonicus* (S. Wright, 1935) and *N. nordestinus* (S. Wright, 1935) that they could be considered as variations of a single polymorphic species. However, this subspecies is currently considered to be a valid taxon (Dussart and Defaye 2002; WoRMS 2019). According to Dussart (1984) the distribution of *N. amazonicus* s.l. includes the Andean, Amazonian, Orinoco-Venezuelan, Guyanean, and the Paranean regions.

The morphological characters discussed by Löffler (1963) seem too vague and incomplete to soundly allow the establishment of a subspecies, and a taxonomic revision of *N. amazonicus* s.l. is desirable. Steinitz-Kannan (1979) reported from Lake El Junco, San Cristobal Island, Galapagos, a calanoid resembling *N. amazonicus*, which was the most abundant zooplanktonic organism in the lake. However, Steinitz-Kannan did not offer drawings or a detailed description that could establish with certainty the identity of this record. Verification of this record is needed.

##### *Notodiaptomus cannarensis* Alonso, Santos-Silva & Jaume, 2017

**Distribution.** Amazon basin, Ecuadorian Andes (Alonso et al. 2017).

**Remarks.** This species is only known from the type locality, the Mazar reservoir on Paute River, Cañar Province, southern Ecuador. The river is eutrophic, belongs to the Amazon basin, and is 2127 m a.s.l. This species is recorded as the most abundant crustacean in the water column of the reservoir, and, considering its restricted known distribution, it is presumably endemic to the region. *Notodiaptomus cannarensis* has a mean length of 1.4 mm and is a remarkable species among calanoid copepods for its symmetrical aliform projections, which are laterally inserted on the female genital somite, and the presence of a conspicuous lamella on the exp-2 of the right P5 in males. All information available to the organism comes from its original description in Alonso et al. (2017).

## Discussion

The identification of European-like species from other parts of the world was a tendency during the nineteenth century (Boxshall and Defaye 2008), and the same applies to the first half of the twentieth century. In the second half of the twentieth century, revisionary studies based on fine-scale taxonomic resolution have recognized numerous species complexes in place of so-called cosmopolitan species (Boxshall and Defaye 2008). Similar to the Cladocera (López et al. 2018a), some species in this checklist may belong to undescribed species or to groups of species with unresolved taxonomic status in the Neotropics and worldwide. For example, *Acanthocyclops robustus*, *Acanthocyclops vernalis*, and *Eucyclops serrulatus* are considered to be cosmopolitan and obviously distributed in the Americas. Further studies may reveal that these species do not occur in the New World or that they have a much more restricted distribution than what has been reported, as for exemple *Eucyclops serrulatus* according to Mercado-Salas et al. (2016). We cannot, however, exclude the possibility that tropical high-altitude aquatic habitats could harbor relict populations originating from northern latitudes (Van Damme and Eggermont 2011) or that some records might be human-mediated introductions (López et al. 2018a).

Our knowledge on the free-living freshwater copepod fauna from continental Ecuador and Galapagos Archipelago, in comparison to other countries in tropical South America, is relatively recent and rather limited. Countries with ecosystem diversity similar to that occurring in Ecuador have their biodiversity much better documented. For example, a checklist of the free-living copepods of the continental waters of Colombia (Gaviria and Aranguren 2007) reported 69 species and subspecies (14 Calanoida, 41 Cyclopoida, and 14 Harpacticoida). Having examined only 38 crustacean samples, Dussart (1984) increased the number of copepod species known to Venezuela from 28 to 66. From a single Colombian coastal lagoon Fuentes-Reinés and Suárez-Morales (2015) reported 15 copepod species, 10 of which typically live or can occur in freshwater. In a study along La Plata basin, Perbiche-Neves et al. (2014b) found 32 cyclopoid species.

Ecuador is a region with high species richness and high rates of endemism (Myers et al. 2000). Dussart (1984) provided a list of the South American copepod species and showed their distribution among the nine biogeographic zones of the continent. By being situated in both the Andean and the Amazonian biogeographic zones, Ecuador might be home to a significant part of the copepod fauna of both regions. Hence, the current low species richness of the Ecuadorian copepod fauna is most likely the effect of the scarce sampling effort rather than a real biogeographic pattern. We expect that geographically large-scale collections that take the extraordinary diversity of the habitats and strong altitudinal gradients in Ecuador into account will reveal a biodiversity at least a magnitude greater than what is currently known for the Copepoda. The recommendations of López et al. (2018a) for more extensive collections of the Cladocera, including specimens suitable for molecular studies, also hold true for the copepods. From among the four lake provinces [Paramo, Andean (under the Paramo, 2000–3500 m a.s.l.), Amazonia, and coastal plains and Andean foothills; Steinitz-Kannan et al. 1983], the lake-poor coastal region might be the greatest challenge to explore, albeit marsh-lakes and ephemeral swamps can harbor rich fauna of copepods (Reid 1987). Special attention must be directed at various altitudinal and latitudinal zones within the country to transitional or cryptic habitats such as littoral zones, temporary pools, mosses, phytotelmata, hyporheic zones, wetlands, cisterns, and other habitats.

A taxonomic and zoogeographic revision of the inland water copepods of Ecuador, using both morphological and genetic information, might allow us to test of some major questions of copepod biogeography and evolution (Table 2). To date, any attempt to infer local as well as broad biodiversity and biogeographic patterns of copepods within Ecuador would be premature due to the scarcity of data, dubious records and unsolved taxonomic problems. A better understanding of the biogeography, biodiversity and phylogenetic relationships of the Ecuadorian fauna, can only be reached if the taxonomic and faunistic data are interpreted within a broad geographic frame. To achieve this goal, we need a network of collaboration, with limnologists and taxonomists from both Ecuador and outside the country.

**Table 2.** Some questions about the biogeography, biodiversity, and evolution of the New World Copepoda that could be answered with extensive taxonomic exploration of the Ecuadorian inland water fauna.

Main topics	Quesions
Dispersal corridor	Might the American Cordillera act as dispersal corridor between North and South America for temperate- or cold-adapted copepods (e.g., see the *Acanthocyclops robustus-vernalis* complex)?
Biogeographical barrier	Are the Andes an insurmountable barrier for the dispersal of lowland/thermophilic copepods (i.e. how does the copepod fauna of the Coastal and Amazonian regions differ from each other)? Comparisons might be made between copepods living in the benthic and in the hyporheic zones of rivers, semiterrestrial and cryptic habitats such as mosses, phytotelmata, forest litter, etc., as well as in temporary collections of water (i.e. ponds, pools and marshes), rather than limnetic copepods, as the coastal region has no natural lakes (Steinitz-Kannan et al. 1983).
Patterns of speciation within islands	Have inland water copepods undergone an evolutionary radiation similar to those found in the terrestrial organisms (Parent et al. 2008) in the Galapagos archipelago, which apparently has a shortage of the fresh surface water bodies (Steinitz-Kannan et al. 1983; López et al. 2018b)? To date, except for the records of the harpacticoids *Cletocamptus axi* and *Cletocamptus schmidti*, the cyclopoids *Eucyclops agilis*, *Microcyclops* sp., and *Metacyclops mendocinus* in Santa Cruz island (Peck 1994), and a calanoid resembling *Notodiaptomus amazonicus* from Lake El Junco in San Cristobal island (Steinitz-Kannan 1979), we have no information on the freshwater copepods of the archipelago.
Dispersal capacity, biodiversity and biogeography	How do the diversity and geographic distributional patterns change in Copepoda with different dispersal ability (Cyclopidae are considered to be good dispersers, while Diaptomidae are poor dispersers; Canthocamptidae are good but most of the Parastenocarididae studied so far seem to be very restricted geographically)?
Diversity and endemism	Are copepods less diverse, but with higher rates of endemism in high altitudinal lakes and rivers?
